# Analgesia in adult trauma patients in physician-staffed Austrian helicopter rescue: a 12-year registry analysis

**DOI:** 10.1186/s13049-021-00839-9

**Published:** 2021-02-01

**Authors:** Christopher Rugg, Simon Woyke, Wolfgang Voelckel, Peter Paal, Mathias Ströhle

**Affiliations:** 1 Department of Anaesthesiology and Critical Care Medicine, Medical University of Innsbruck, Anichstrasse 35, 6020 Innsbruck, Austria; 2 Department of Anaesthesiology and Intensive Care Medicine AUVA Trauma Centre Salzburg, Academic Teaching Hospital of the Paracelsus Medical University, Dr.-Franz-Rehrl-Platz 5, 5010 Salzburg, Austria; 3 Department of Anaesthesiology and Intensive Care Medicine, Hospitallers Brothers Hospital, Paracelsus Medical University, Kajetanerplatz 1, 5010 Salzburg, Austria; 4 Austrian Society for Mountain and High-altitude Medicine (ÖGAHM), Lehnrain 30a, 6414 Mieming, Austria

## Abstract

**Background:**

Sufficient analgesia is an obligation, but oligoanalgesia (NRS> 3) is frequently observed prehospitally. Potent analgesics may cause severe adverse events. Thus, analgesia in the helicopter emergency medical service (HEMS) setting is challenging. Adequacy, efficacy and administration safety of potent analgesics pertaining to injured patients in HEMS were analysed.

**Methods:**

Observational study evaluating data from 14 year-round physician-staffed helicopter bases in Austria in a 12-year timeframe.

**Results:**

Overall, 47,985 (34.3%) patients received analgesics, 26,059 of whom were adult patients, injured and not mechanically ventilated on site. Main drugs administered were opioids (*n*=20,051; 76.9%), esketamine (*n*=9082; 34.9%), metamizole (*n*=798; 3.1%) and NSAIDs (*n*=483; 1.9%). Monotherapy with opioids or esketamine was the most common regimen (*n*=21,743; 83.4%), while opioids together with esketamine (*n*= 3591; 13.8%) or metamizole (*n*=369; 1.4%) were the most common combinations. Females received opioids less frequently than did males (*n*=6038; 74.5% vs. *n*=14,013; 78.1%; *p*< 0.001). Pain relief was often sufficient (> 95%), but females more often had moderate to severe pain on arrival in hospital (*n*=34; 5.0% vs. *n*=59; 3.2%; *p*=0.043). Administration of potent analgesics was safe, as indicated by MEES, SpO_2_ and respiratory rates. On 10% of all missions, clinical patient assessment was deemed sufficient by HEMS physicians and monitoring was spared.

**Conclusions:**

Opioids and esketamine alone or in combination were the analgesics of choice in physician-staffed HEMS in Austria. Analgesia was often sufficient, but females more than males suffered from oligoanalgesia on hospital arrival. Administration safety was high, justifying liberal use of potent analgesics in physician-staffed HEMS.

**Supplementary Information:**

The online version contains supplementary material available at 10.1186/s13049-021-00839-9.

## Introduction

Providing sufficient analgesia in the challenging prehospital emergency setting is an ethical obligation and of clinical relevance [1]. Potent analgesics, able to relieve intense pain, can result in severe adverse events (e.g. respiratory or circulatory depression, hallucination or agitation). Thus, their liberal use is limited [2, 3]. In the special stetting of helicopter emergency medical services (HEMS) severely injured patients needing analgesia are frequent. Physicians must be aware that the handling of severe side-effects during HEMS operations is difficult in a hostile environment with limited in-cabin space. Adequacy, efficacy and safety of prehospital analgesia administered by HEMS have been addressed before, often with reports on high rates of oligoanalgesia [4–8]. The aim of this study was to assess analgesia in patients treated by HEMS physicians over a 12-year timeframe in Austria. Dosages and analgesic regimens were examined, as were adequacy and safety of administration.

## Materials and methods

This retrospective study was approved by the Ethics Committee of the Medical University of Innsbruck (AN2015–0068 347/4.13393/5.20) and was registered with Clinical Trials (NCT03760302). In Austria the ÖAMTC (Austrian Automobile, Motorcycle and Touring Club) runs 17 year-round helicopter bases. The HEMS team consists of a pilot, a physician (advanced life support (ALS)-certified with several years of clinical practice, most commonly in anaesthesiology and intensive care medicine) and an emergency medical technician with basic life support and mountain rescue skills. After completing a rescue mission, emergency physicians transfer data from their handwritten medical report to a standardized digital database intended for medical documentation and billing purposes. Analysis of this database was performed for a 12-year timeframe from 01/01/2006 to 31/12/2017. Two helicopter bases were excluded for reasons of data protection restrictions, and one helicopter program was launched in May 2020. Consequently, this analysis drew on nationwide data from 14 year-round helicopter bases. The flow chart for the study is outlined in Fig. A1.

Data obtained included date, time, helicopter base, type of accident or emergency, sex, age in groups, injury pattern, severity and region of injuries, medication and interventions performed by the emergency team. With regard to emergency classifications, *mountain accidents* were analysed separately.

The largest group of *other accidents* included work, road traffic and home and leisure accidents. Pain was scaled as *no pain*, *mild pain* (Numeric Rating Scale (NRS) ≤3), and *moderate to severe pain* (NRS > 3). With regard to analgesics, *opioids* mainly included fentanyl, piritramide, morphine and also seldomly tramadol, nalbuphine, sufentanil and remifentanil. *NSAIDs* administered included aspirin, mefenamic acid, diclofenac and ketoprofen.

Age documentation was performed in 5- to 10-year scales. With regard to expected weight and required dosages, patients at least 15 years of age were enrolled in the study. When medication dosage was missing or not given accurately, only the type of medication was analysed. Severity and progress of the patient’s condition were evaluated using NACA (National Advisory Committee for Aeronautics) scoring and the voluntarily documented MEES (Mainz Emergency Evaluation Score including four categories for Glasgow Coma Scale, heart and breathing rate, cardiac rhythm, pain, blood pressure and peripheral oxygen saturation (SpO_2_)) [9, 10].

Data are presented as median and interquartile range or count and percentage, as appropriate. The Chi-square test was performed to detect group differences in frequencies, and the Mann-Whitney U test for group differences in continuous data. Data were stored with Excel 2019 (Microsoft, Seattle, WA, USA) and processed with RStudio version 1.2.5001 (RStudio, Inc., Boston, MA, USA).

## Results

### Demographics and general emergency characteristics

A total of 176,056 HEMS operations were analysed. Primary missions made up 139,831. Of these, analgesics were administered in 47,985 (34.3%) cases. Exclusion of patients under 15 years of age, uninjured or mechanically ventilated on site resulted in 26,059 patients for further analysis (Fig. A1). Sex was analysed separately, resulting in 17,950 males and 8103 females. General emergency characteristics are depicted in Table 1.

**Table 1:** General emergency characteristics

	Adults (≥ 15 yrs)*n*= 26,059^*^n (%)	
	Male ***n***= 17,950 (68.9)	Female ***n***= 8103 (31.1)
Age (years)		
*15–19*	1679 (9.4)	793 (9.8)
*20–39*	5916 (33.0)	1940 (23.9)
*40–59*	6788 (37.8)	2912 (35.9)
*60–79*	3180 (17.7)	1799 (22.2)
*> 80*	387 (2.2)	659 (8.1)
Emergency classification		
*Accident (mountain)*	5063 (28.2)	3265 (40.3)
*Accident (other)*	12,444 (69.3)	4531 (55.9)
*Other*	443 (2.5)	306 (3.8)
Analgesics used		
*Opioids*	14,013 (78.1)	6038 (74.5)
*Esketamine*	6144 (34.2)	2938 (36.3)
*Metamizole*	516 (2.9)	282 (3.5)
*NSAIDs*^*+*^	323 (1.8)	160 (2.0)
NACA Score as median (IQR)	4 (3–4)	3 (3–4)
Injury localizations per patient		
1	9776 (54.5)	5089 (62.8)
2–3	7072 (39.4)	2645 (32.6)
> 3	1065 (5.9)	356 (4.4)
Injury localizations		
*Head*	4901 (27.3)	1858 (23.0)
*Spine*	4228 (23.6)	1729 (21.3)
*Chest*	4423 (24.6)	1403 (17.3)
*Abdomen*	1537 (8.6)	546 (6.7)
*Pelvis*	1023 (5.7)	411 (5.1)
*Upper limb*	7022 (39.1)	2479 (30.6)
*Lower limb*	8011 (44.6)	4367 (53.9)
Injury types		
Fracture	12,791 (71.3)	6054 (74.7)
Contusion	5930 (33.0)	2424 (29.9)
Soft tissue	6026 (33.6)	1933 (23.9)
Traumatic brain injury	2871 (16.0)	1065 (13.1)
Internal organs	1125 (6.3)	379 (4.7)
Neurovascular	1055 (5.9)	251 (3.1)

* sex unknown in n=6 cases; ^+^ Non-steroidal anti-inflammatory drugs

The adult age group most affected by frequency was the 40- to 59-year-olds. General injury localizations as well as number of affected body regions per patient are depicted in Table 1. With respect to injury severity, the median National Advisory Committee for Aeronautics (NACA) score was 4 (3–4) for males and 3 (3–4) for females, with a total of 12,239 (47.0%) patients suffering potentially life-threatening injuries as classified by a NACA score ≥ 4.

### Analgesics commonly used

**Fig. 1:**
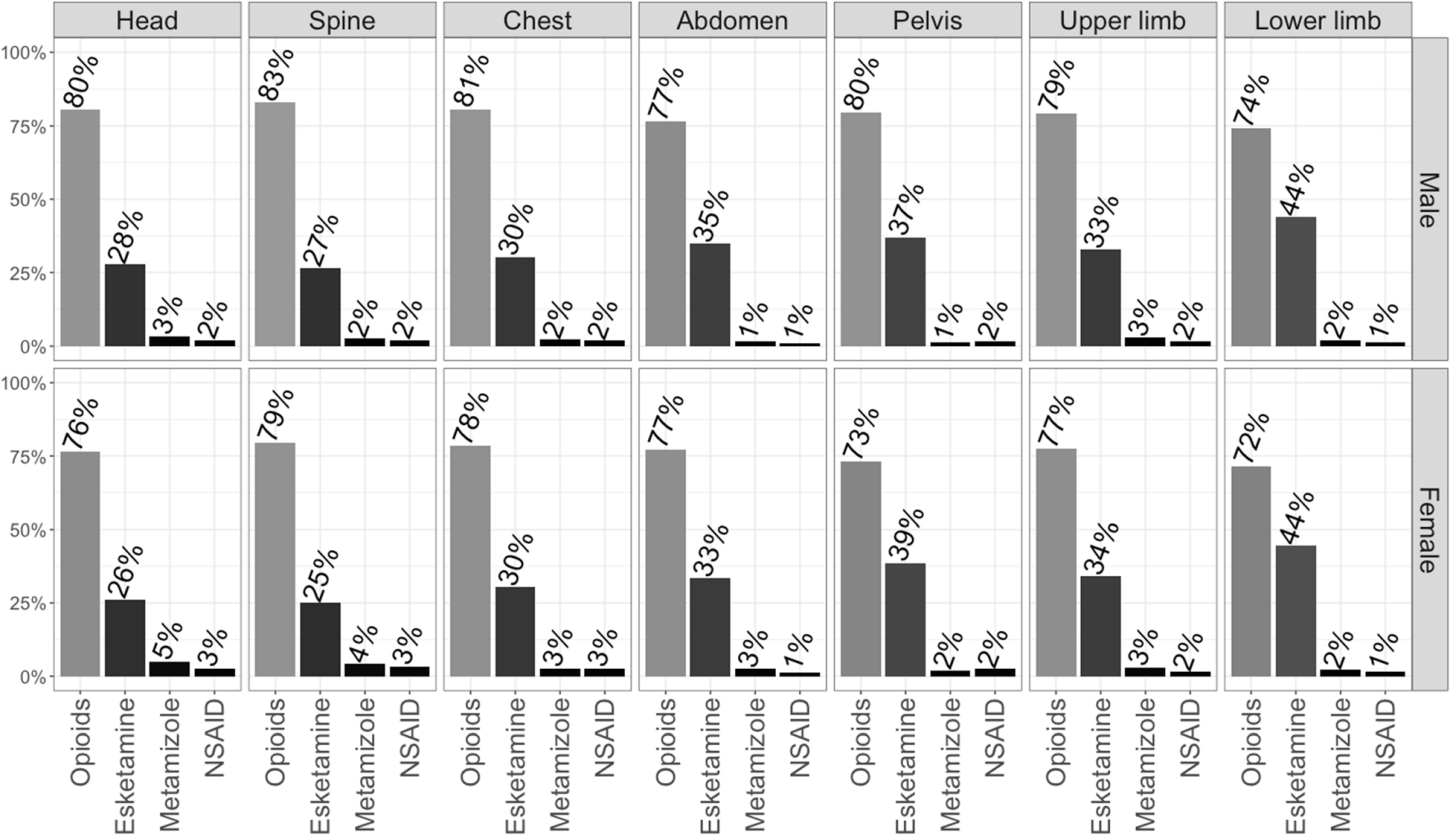
Analgesics commonly used in injured patients in dependency on injury localization. NSAID: Non-steroidal anti-inflammatory drug

Analgesics commonly used are presented as frequencies and median dosages in Table 2. Figure 1 presents analgesics administered dependent on injury localization, and Table 3 shows analgesic regimens in terms of number of analgesic substance classes administered and commonly used combinations.

**Table 2:** Commonly used analgesics in HEMS operations presented as frequencies and median dosage (IQR)

	Adults (≥ 15 yrs)*n*= 26,059^*^	
	Male ***n***= 17,950	Female n= 8103
Fentanyl		
*n (%) total*	8439 (47.0)	3289 (40.6)
*n (%) with dosage*	4267 (50.6)	1706 (51.9)
*Dosage (mg)*	0.20 (0.15–0.25)	0.20 (0.10–0.25)
Piritramide		
*n (%) total*	5244 (29.2)	2559 (31.6)
*n (%) with dosage*	2549 (48.6)	1420 (55.5)
*Dosage (mg)*	7.50 (7.50–15.00)	7.50 (7.50–10.00)
Morphine		
*n (%) total*	384 (2.1)	195 (2.4)
*n (%) with dosage*	112 (29.2)	63 (32.3)
*Dosage (mg)*	10.00 (5.00–10.00)	5.00 (5.00–10.00)
Esketamine		
n (%) total	6144 (34.2)	2938 (36.3)
n (%) with dosage	2076 (33.8)	1123 (38.2)
Dosage (mg)	25.0 (20.0–50.0)	25.0 (15.0–40.0)

* sex unknown in n= 6 cases

**Table 3:** Analgesic therapy regimen for injured patients in HEMS operations

	Adults (≥ 15 yrs)n= 26,059^*^	
	Male n= 17,950	Female n= 8103
Analgesic substance classes		
*Monotherapy*	14,938 (83.2)	6805 (84.0)
*Dual therapy*	2972 (16.6)	1278 (15.8)
*Triple therapy*	40 (0.2)	19 (0.2)
Common combinations		
*Opioids only*	11,056 (61.6)	4781 (59.0)
*Esketamine only*	3539 (19.7)	1818 (22.4)
*Opioids + esketamine*	2520 (14.0)	1071 (13.2)
*Opioids + metamizole*	260 (1.4)	109 (1.3)
*Metamizole only*	199 (1.1)	133 (1.6)
*NSAIDs*^*+*^ *only*	141 (0.8)	72 (0.9)
*Opioids + NSAIDs*^*+*^	136 (0.8)	57 (0.7)

* sex unknown in n= 6 cases; ^+^ Non-steroidal anti-inflammatory drugs

In males, opioids were administered in 78.1%, esketamine in 34.2%; in females, opioids were administered in 74.5% (*p*< 0.001), esketamine in 36.3% (*p*= 0.002) of all cases (Table 1). Sorted by frequency of administration, opioids included mainly fentanyl, piritramide and morphine (Table 2). Opioids were given in a high percentage of patients regardless of injury localization (Fig. 1). Additional or alternative administration of esketamine was performed less often for head or spine injuries and was more pronounced in pelvic and lower limb injuries. Regarding analgesic combination therapies, 83.2% of male and 84.0% of female patients received one, 16.6 and 15.8% two and merely 0.2% of female and male patients were given three different substance classes (Table 3). While approximately 81% of patients (regardless of sex) received opioids or esketamine only, the most common combinations were opioids with esketamine (males: 14.0%; females: 13.2%), opioids with metamizole (1.4%; 1.3%) and opioids with NSAIDs (0.8%; 0.7%). Median doses were 0.2 mg of fentanyl, 7.5 mg of piritramide, 5 mg or 10 mg of morphine and 25 mg or 30 mg of esketamine.

### Adequacy and administration safety of potent analgesics

The following analysis was performed on patients with complete datasets, including documentation on site and on arrival in hospital, leaving 2330 patients for analysis of respiratory items of MEES documentation and 2517 patients for analysis of pain scores.

**Fig. 2:**
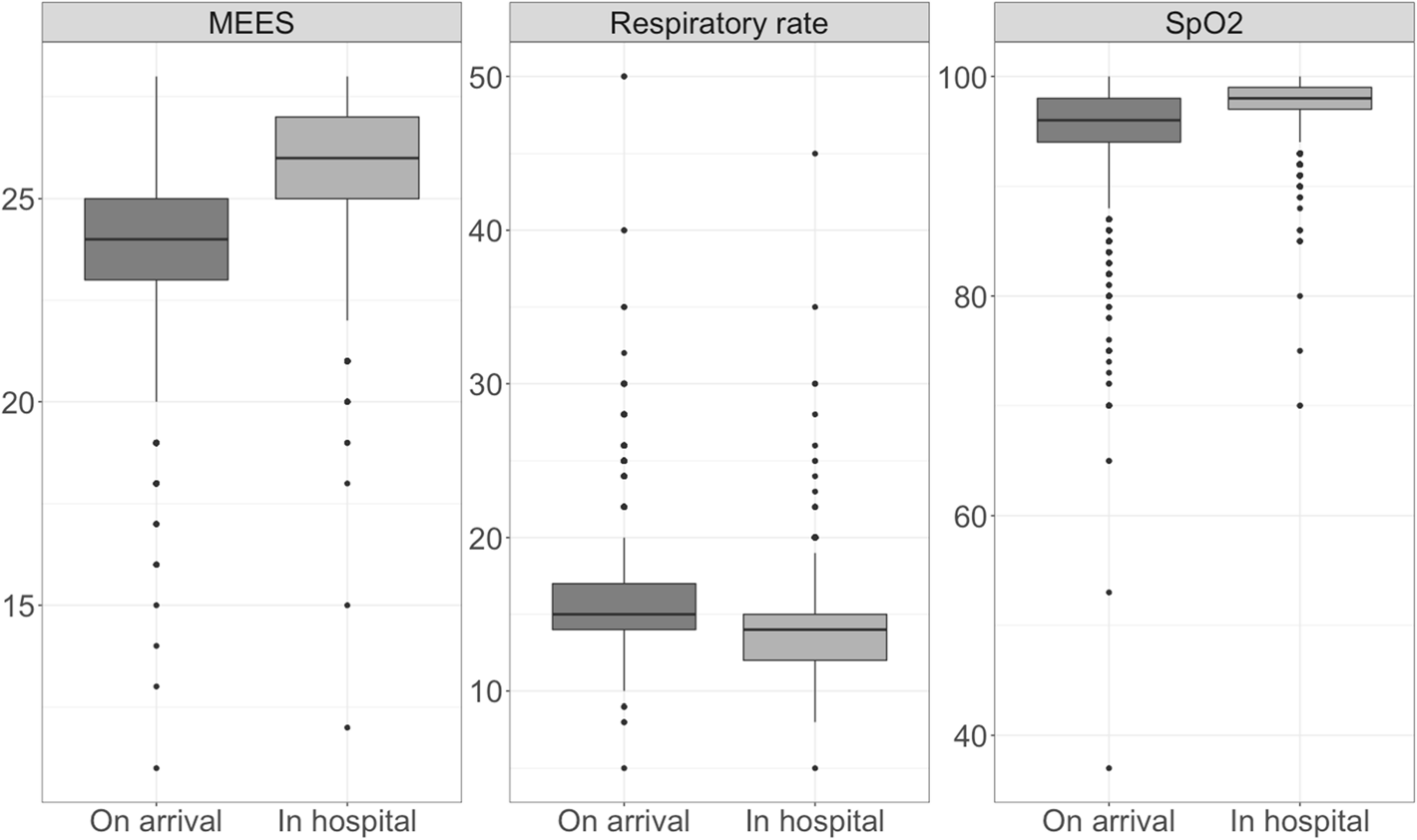
MEES, respiratory rate and SpO_2_ of injured patients receiving medical analgesia on arrival of emergency physician compared to at handover in hospital. Only patients with complete data were included (*n*= 2330). Due to missing differences, a gender independent presentation was chosen

Figure 2 illustrates differences in MEES, respiratory rates and SpO_2_ in injured adults between the time when the emergency physician arrives on site and the time when the patient is handed over to the hospital. MEES increased from 24 (23–25) to 26 (25–27) (*p*< 0.001). Respiratory rates decreased from 15 (14–17) to 14 (12–15) (p< 0.001). Oxygen saturation improved from 96 (94–98) to 98 (97–99) (p< 0.001). Additional mechanical ventilation requirements during transport were present in only 45 (0.2%) patients.

**Fig. 3:**
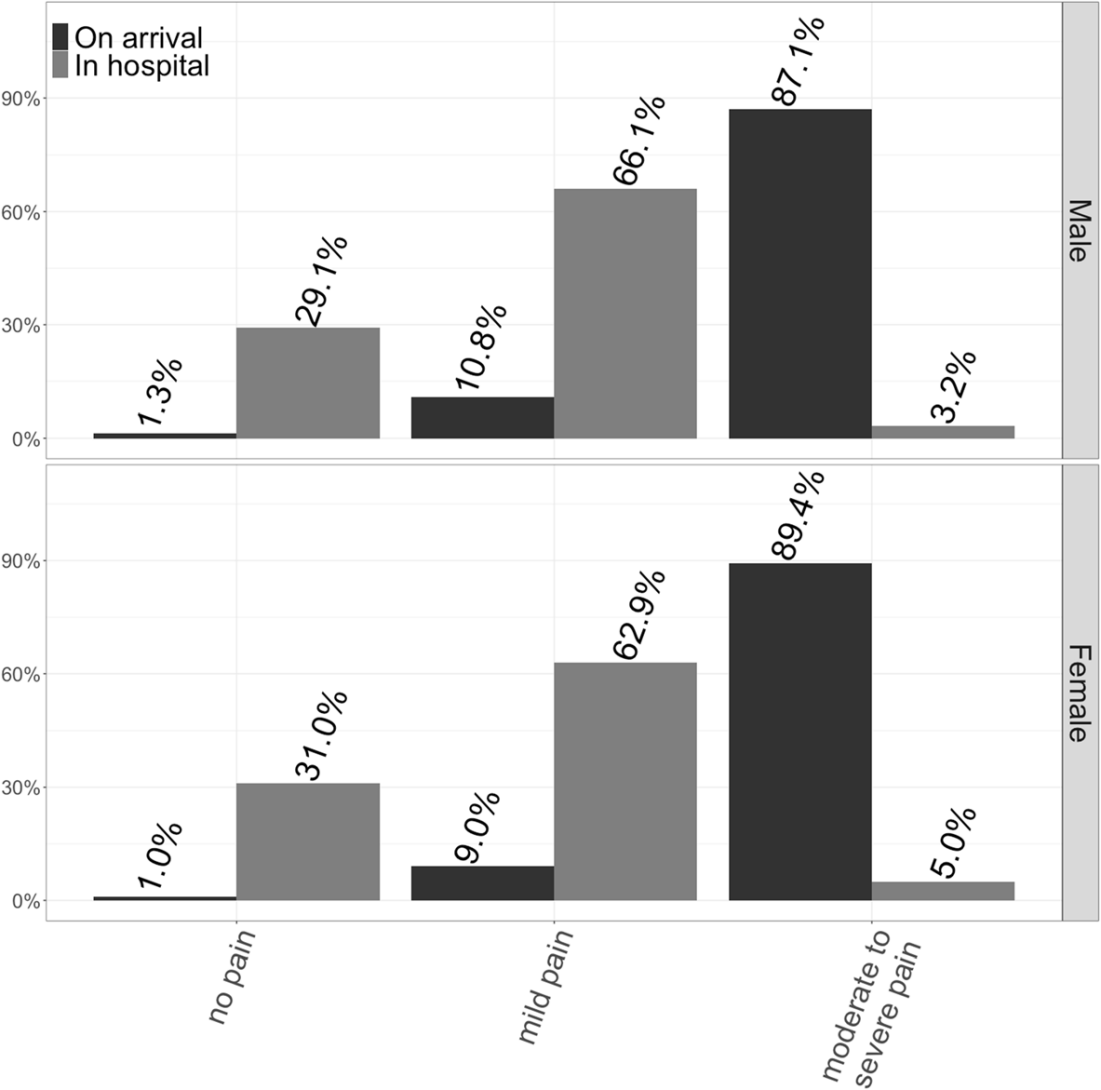
Pain level of injured patients receiving medical analgesia on arrival of emergency physician compared to at handover in hospital. Only patients with complete data regarding level of pain were considered (*n*= 1840 male adults; *n*= 677 female adults)

At the same time, documented pain decreased with respect to the two observed timepoints (Fig. 3). Regarding gender differences, no significant difference was detected in patients with moderate to severe pain on arrival of the emergency physician (male: 87.1% vs. female: 89.4%; *p*= 0.137), but after treatment and on arrival at hospital females more often suffered from moderate to severe pain (male: 3.2% vs. female: 5%; *p*= 0.043). The fraction suffering no or mild pain increased significantly.

We identified 2723 missions (10%) conducted without technical monitoring such as pulse oximetry, ECG or blood pressure. The majority of patients were male (*n*=1724; 63.3%), younger (15-39a: *n*=1160; 42.6%), orientated (*n*=1877; 91.9%) and with moderate to severe pain (*n*=2393; 87.9%) from fractures (*n*=2199; 80.8%) to the upper or lower limb (*n*=2466; 90.6%). Regarding emergency classifications, 2023 (74.3%) cases were mountain accidents and 1877 (68.9%) occurred between December and May. Opioids were administered to 2071 (76.1%) patients and esketamine to 913 (33.5%). Median dosages were 0.2 mg fentanyl, 7.5 mg piritramide and 25 mg esketamine.

## Discussion

This study focuses on emergency patients > 15 years of age, nearly half of whom received analgesic therapy in a potentially life-threatening condition (NACA > 3). Injuries were predominantly fractures, with the upper or lower extremities most often affected. Main substances administered were opioids (fentanyl > piritramide > morphine) followed by esketamine, metamizole and NSAIDs. With analgesic monotherapy being the most common regimen, males proportionally received opioids more often than did females. Following opioids or esketamine only, opioids with esketamine or opioids with metamizole were the most common combination therapies. As shown by the development of MEES, respiratory rates, oxygen saturation and the non-necessity of mechanical ventilation during transport, application of potent analgesics was very safe in the described setting. A total of 10% of the analysed HEMS operations were performed without technical monitoring which could be due to various reasons like short transportation time, cold weather and winter clothes, technical rescue operations or even patient’s medical condition. Regarding pain relief, a higher fraction of females still had moderate to severe pain on hospital arrival. In total, pain levels decreased significantly, and administration seemed safe, thus justifying the liberal use of potent analgesics in HEMS.

This study presents a large retrospective analysis of a nationwide registry covering 14 physician-staffed helicopter bases in Austria over a 12-year timeframe. To date, only few studies of this size have been published on prehospital analgesia in HEMS.

### Demographics and general emergency characteristics

Current publications on analgesia in HEMS originate in the combat setting, in particular with the U.S. armed forces in Afghanistan [11–14]. Affected patients were primarily young male soldiers who suffered acute trauma due to blast or penetrating injuries. Civilian HEMS operations report data from Europe [3–6, 15, 16] and also Australia [8]. The data analysed show that 34.3% of patients received analgesic therapy. This compares with the figures reported for HEMS operations in Victoria, Australia (89%) [8], Germany (79%) [4], Switzerland (57–84%) [15, 17]. Regarding ground-based EMS, two studies from Europe reported a percentage of 48% of all patients [2, 3], whereas merely 3% of all trauma patients in a South African study received any kind of analgesic medication [18]. As shown in other studies conducted in Austria, a high density of HEMS in combination with a mountainous landscape often prompts airborne rescue of merely mildly or even non-injured patients [19, 20]. This can also be seen from our data, where a total of 44.6% of all HEMS operations were classified as NACA ≤ 3. This may explain the rather small percentage of patients receiving analgesia in this study.

### Analgesics commonly used

Analgesia administered during EMS may vary depending on type (HEMS/ EMS) and staffing (e.g. paramedic vs. physician) including the training level of the providers (anaesthesiologist vs. general practitioner, specialist vs. trainee, BLS vs. ALS provider, clinical experience). In systems with a large volume of emergency operations staffed at most with BLS/ILS providers, administration of i.v. analgesics is limited [18]. Nitrous oxide can be an option, but is mainly used in some English-speaking countries [18]. Another inhalational analgesic is low-dose methoxyflurane [21, 22]. Despite its undeniable advantages, as in quick, easy and safe administration also by non-physicians, its distribution seems very limited up to date (e.g. Australia, Italy). Moreover, as also confirmed in this study, opioids and ketamine/ esketamine predominate in the prehospital management of moderate to severe pain [2–5, 11–14]. Whether racemic ketamine or its S-enantiomer esketamine is preferred depends mainly on country-specific regulations. When comparing the two, consideration of different dosing requirements is important. As shown in a study on analgesia in HEMS in Switzerland, ketamine was preferably used by anaesthetists [17]. The high proportion of Austrian emergency physicians also being anaesthetists also explains the rather high rate of esketamine use in this study. In detail, the presented data were able to show high use of opioids over all injury localizations and the predominant use of esketamine in injuries affecting the upper and lower limbs including the pelvis. Fortunately, the administration of these potent analgesics is not limited to physicians, at least in some countries. Albeit median doses are somewhat higher in this physician-staffed service, paramedics can effectively and safely administer opioids and ketamine [8, 23–27]. Furthermore, dose differences are not only present inter- but also intra-professionally as demonstrated by a study from a physician-staffed EMS in Germany, which showed differences in pain treatment between surgeons and anaesthesiologists, particularly regarding opioids [28]. Ketamine has been described as being safe and effective alone – even as effective as an alternative opioid – and also as being able to reduce opioid requirements when used in combination with opioids [26, 29, 30]. After opioids or esketamine administered alone, the by far most commonly administered combination therapy in the presented study was an opioid with esketamine, followed by a combination of an opioid with metamizole.

### Adequacy and application safety of potent analgesics for injured patients during HEMS operations

Moderate to severe pain is a frequent finding in the prehospital care of emergency patients [8, 17, 31]. So is the rate of inadequate pain treatment, also described as oligoanalgesia (18–58%) [4–6, 24].

While the absence of analgesic administration as well as a higher NACA Score and NRS on site have unsurprisingly been described as risk factors for insufficient pain management, the same is unexpectedly also true of treatment by a female physician [6]. Although perceived oligoanalgesia rates were the same, emergency physicians improved quality of analgesia by providing a substantially higher NRS reduction than did paramedics in a study from Switzerland [2]. As higher doses of fentanyl administered in a paramedic setting have been shown to relieve pain better [23], the observed benefits might be due to deliberately increased dosages in physician-staffed settings, as also seen in this study. Ketamine has been shown to be safe, when administered alone or in combination with opioids, with no loss of consciousness, oxygen desaturation or clinically significant emergence reactions occurring [7, 27, 30]. Ketamine alone seems to have fewer side-effects than morphine alone, but the combination of both has more side-effects than morphine alone [32]. Albeit a commonly used combination therapy, the administration of ketamine with morphine has also not been recommended because of uncertainties regarding safety [32, 33]. Adverse events due to analgesic medications are not easy to discriminate in an emergency and sometimes austere situation. With regard to potent analgesics as in opioids or esketamine, the most feared and clinically important side-effect is certainly a possible respiratory depression. Comparison of the need for additional mechanical ventilation during transport as well as clinical scores (MEES), surrogates for sufficient respiration (SpO_2_, respiratory rate) and levels of pain on arrival of the emergency physician and at the time of handover in hospital led us to conclude that the analgesics administered in this study were safe and adequate. The percentage of patients suffering from moderate to severe pain unmistakably decreased from over 87% to under 5%, further justifying liberal use of potent analgesics. Interestingly, while no gender differences were recorded with respect to initially moderate to severe pain, females more often still suffered from moderate to severe pain on arrival in hospital. Documented injury severity was lower in females and, while median analgesic dosages were comparable, decreased opioid but increased esketamine administration was recorded in female adults. Reasons for this difference cannot be derived from the presented data, but this finding stands in contrast to the existing literature, where largely no gender difference or even a female predominance in pain relief is described [2, 4–6, 18].

Not previously described is the fact that 10% of the described HEMS missions were conducted without any technical monitoring despite the overwhelming use of opioids and esketamine. These special cases were particularly young, orientated men involved in mountain accidents mainly in winter and suffering from severe pain from fractures to the upper or lower limbs. Obviously, this practice is not uncommon and emergency physicians were not discouraged from administering these potent analgesics in order to relieve severe pain despite the non-availability or cold-related failure of adequate technical monitoring equipment.

### Limitations

Although handwritten report forms were primarily documented prospectively and transferred to the digital database in a timely manner and by the emergency physician himself, poor documentation quality is not uncommon in emergency prehospital settings [23, 34]. Errors occurring during data transfer might have additionally contributed to this problem. Accurate documentation has been proposed as a quality indicator of physician staffed emergency medical service [35] but the mean proportion of completely documented cases often remains low, as seen in a recent Nordic study on HEMS [36]. Comparing to proportions of 25–91% reported by them, merely 9–10% of all cases in this study were completely documented, including MEES, SpO2-values, respiratory rates and pain levels from initial on-site evaluation and hospital admission. A reporting bias can therefore not be excluded. Furthermore, pain levels were documented with an NRS-guided scale and not with exact numerical documentation. Detailed analysis of pain reduction (e.g. NRS reduction) was therefore not possible. Furthermore, data analysis in general was conducted retrospectively.

## Conclusion

Opioids and esketamine were frequently administered in physician-staffed HEMS. Analgesia was largely sufficient, with females more often suffering from oligoanalgesia (NRS> 3) on hospital arrival. Administration safety was high, justifying liberal use of potent analgesics in physician-staffed HEMS.

## Supplementary Information

**Additional file 1 Figure A1.** Consort Flowchart.

## Data Availability

No data are available. Participant data from the ÖAMTC CFV. All data are deidentified. The data set was delivered containing only serial numbers for each participant. Protocol and statistical analysis plans are available.
