# Supplementary figures and images for: Analgesia in adult trauma patients in physician-staffed Austrian helicopter rescue: a 12-year registry analysis
Source: Scand J Trauma Resusc Emerg Med. 2021 Feb 1;29:28. doi: 10.1186/s13049-021-00839-9 (PMC7852148; doi:10.1186/s13049-021-00839-9)

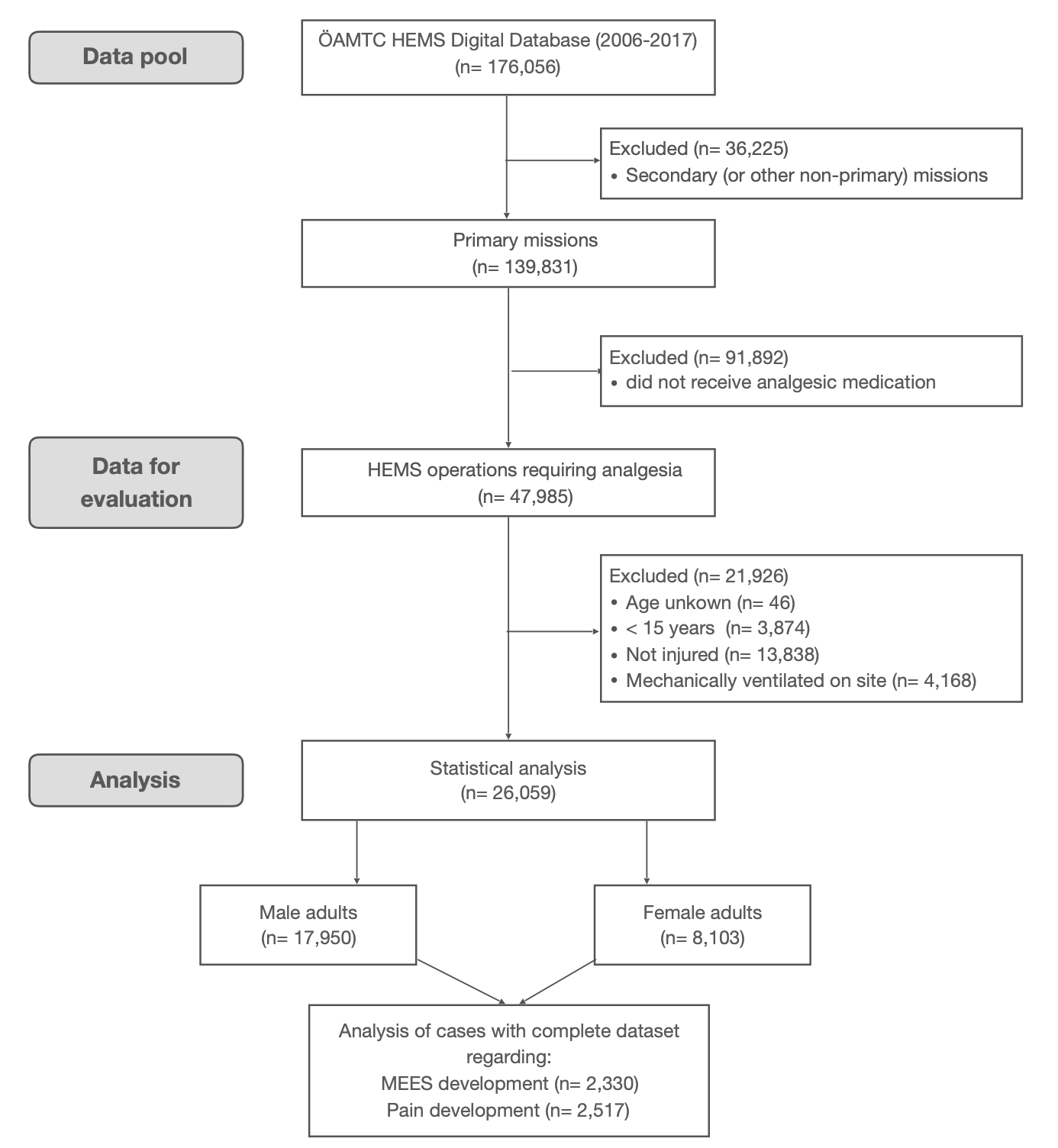

Supplement: Supplementary file 1 — Additional file 1 Figure A1. Consort Flowchart. [file 13049_2021_839_MOESM1_ESM.png]
